# Impact of Introduction Timing on Home-Visit Rehabilitation Outcomes for Older Adults Supported by the Japanese Nursing Care Insurance System

**DOI:** 10.7759/cureus.94092

**Published:** 2025-10-08

**Authors:** Yuji Nakagawa, Akio Goda, Yoshinori Maki, Daisuke Yoshino, Masayuki Sakurai

**Affiliations:** 1 Rehabilitation, Hikari Hospital, Otsu, JPN; 2 Physical Therapy, Faculty of Health and Medical Sciences, Hokuriku University, Kanazawa, JPN; 3 Neurosurgery, Ayabe Renaiss Hospital, Ayabe, JPN; 4 Neurosurgery, Hikone Chuo Hospital, Hikone, JPN

**Keywords:** activities of daily living, clinical outcomes, frenchay activities index, home-visit rehabilitation, initiation timing, instrumental activities of daily living, nursing care insurance

## Abstract

Background

In the aging Japanese society, home-visit medical and nursing services are becoming essential for the elderly population. Home-visit rehabilitation is a service available based on the nursing care insurance system in Japan. However, little is known about the association between home-visit rehabilitation introduction timing (during in-home life or soon after discharge from a hospital or a nursing facility) and clinical outcomes for older adults. This study aimed to examine the association between initiation timing and clinical effectiveness.

Method

This exploratory retrospective study included 212 older adults who received home-visit rehabilitation using nursing care insurance from November 2018 to April 2023. Participants were divided into two groups: those who initiated home-visit rehabilitation while living at home (Group A) and those who started it soon after being discharged from a hospital or a nursing facility (Group B). Clinical variables were collected, and functional outcomes were compared between the two groups using the Barthel Index (BI), Frenchay Activities Index (FAI), levels of support or long-term care needed, degree of being bedridden, and dementia rating. Statistical significance was defined as a p-value < 0.05.

Results

Compared to older adults in group A, those in group B had a higher care level when home-visit rehabilitation began. The degree of independence in daily living and FAI scores were significantly lower in Group B. The frequency of home-visit rehabilitation was relatively high in the same group. The changes in BI and FAI scores before and after the initiation of home visit rehabilitation and the proportion of older adults who maintained or improved their daily life independence rank were significantly higher in group B.

Conclusions

Frequent home-visit rehabilitation initiated soon after hospital discharge was associated with the maintenance and improvement of activities of daily living (ADLs) and instrumental activities of daily living (IADLs) in older adults, although this association is likely confounded by higher rehabilitation intensity in the post-discharge group.

## Introduction

In response to Japan’s aging population, the government has promoted a community-based integrated care system that enables older adults to continue living in their familiar environments, even after developing a need for support or nursing care services [1]. This system comprehensively covers life support, housing, nursing care, and medical prevention and treatment. To enable older adults to continue living in their own communities, it is essential to improve, or at least maintain, their activities of daily living (ADLs) and instrumental activities of daily living (IADLs). Home-visit rehabilitation is a service covered by the Japanese nursing care insurance system that aims to improve or maintain the ADLs and IADLs of older adults [1].

Home-visit rehabilitation provides essential therapeutic services, such as physical and occupational therapy, delivered at home to enhance or sustain the physical and mental capacities of individuals requiring in-home care, thereby promoting independent living [1]. The number of older adults receiving such rehabilitation services at home is increasing [1], and it has been shown to improve not only ADLs and IADLs but also life satisfaction [2]. Wu et al. reported that home-visit rehabilitation was effective in improving physical function in older adults after hip fracture [3]. Kamioka et al. found that ADLs improved in older adults who received home-visit rehabilitation within one year after disease onset or injury [4], while Ishida et al. demonstrated IADL improvements in older adults receiving home-visit rehabilitation for over one year [5]. These findings suggest that home-visit rehabilitation plays an essential role in supporting older adults living at home.

However, to the best of our knowledge, clinical evidence regarding the optimal timing of home-visit rehabilitation initiation remains limited. While the general benefits of this service are well-documented, less is known about how outcomes differ between patients who start rehabilitation proactively while living at home versus those who start immediately following a hospital or facility discharge. Furthermore, how the timing of initiation interacts with the intensity of rehabilitation in a real-world clinical setting is not well understood.

Therefore, the present exploratory study aimed to describe and compare the clinical characteristics and functional outcomes of older adults based on two different timings of home-visit rehabilitation initiation. Rather than determining causality, our goal was to provide a real-world snapshot of current clinical practice and to generate hypotheses for future, more rigorous research on this topic.

## Materials and methods

Study design

This retrospective study included 212 older adults who received home-visit rehabilitation therapy under Japan’s nursing care insurance system at Hikari Hospital between November 2018 and April 2023. Participants were identified by searching the hospital's electronic medical records for all patients who received home-visit rehabilitation under the nursing care insurance system during the study period. All patients meeting these initial criteria were included in the study, provided their records contained complete data for the variables of interest, including baseline and follow-up assessments of the Barthel Index and Frenchay Activities Index. Patients with incomplete primary outcome data were excluded. To examine whether the timing of initiating home-visit rehabilitation affects functional outcomes, participants were divided into two groups based on the initiation timing: those who began home-visit rehabilitation while living at home (Group A) and those who began it soon after being discharged from a hospital or nursing facility (Group B). Clinical data were collected and compared at two time points: at the initiation of home-visit rehabilitation and either six months later or at the time of termination. For participants who discontinued home-visit rehabilitation within six months, the most recent Barthel Index (BI) and Frenchay Activities Index (FAI) scores at the time of termination were used in the analysis. This study was approved by the ethics committee of Hikari Hospital (approval number: 2025-1).

Data collection

Baseline Characteristics

Baseline characteristics were obtained from medical records, including sex, age, underlying conditions for home-visit rehabilitation (malignancy/cancer, motor function disease, respiratory disease, cardiovascular disease, cerebrovascular disease, disuse syndrome, neuromuscular disease, visceral disease, dementia, and mental illness), and information about cohabitation (spouse and/or relatives). The frequency of home-visit rehabilitation (once a week or more) and session duration (40 or 60 minutes) were also recorded. The frequency and duration of rehabilitation sessions were determined by the attending physician and therapist based on the patient's clinical needs and goals, in accordance with the limits of the nursing care insurance system. While the exact frequency varied, it was categorized as either 'once a week' or 'two or more times per week' for the purpose of this analysis. Patients’ physical, mental, and functional statuses were assessed using the Barthel Index (BI), the Japanese version of the Frenchay Activities Index (FAI), and official ratings for bedridden status and dementia severity.

Functional Assessments (ADL/IADL)

Barthel Index (BI):** **BI [6] is used to assess basic activities of daily living (ADLs). It consists of 10 items, including feeding, bathing, grooming, dressing, toileting, and mobility-related tasks such as transferring and stair climbing. Each item is scored with 0, 5, 10, or 15 points. The total score ranges from 0 to 100.

Frenchay Activities Index (FAI):** **FAI [7] is a measure of instrumental activities of daily living (IADLs), composed of 15 items that reflect the frequency of engagement in daily tasks. These tasks include preparing meals, washing dishes, doing laundry, performing both light and heavy housework, and shopping for daily necessities. The scale also evaluates social participation (e.g., attending social events, walking outdoors for more than 15 minutes, engaging in hobbies), transportation use (e.g., driving, using public transportation, going on outings), and other activities such as gardening, maintenance, reading, and paid employment. Each item is scored on a four-point scale (0 to 3), and the total score ranges from 0 to 45.

Certification of Care Level and Functional Classifications

The definition of levels of support or long-term care needed and the degree of being bedridden and dementia rating in Japan were detailed in our previous study [8].

Support or Care-Need Levels

In Japan, long-term care insurance provides services to older adults certified as requiring either support (2 levels) or long-term care (5 levels). Certification is based on two assessments: (a) a physician’s evaluation of the patient’s physical and mental status and (b) an assessment by a long-term care certification committee, comprising professionals in medicine, health, and welfare. A committee member interviews the older adult and their caregivers to finalize the care-need level.

Bedridden and Dementia Ratings

Bedridden status is categorized into grades J, A, B, and C, each with two subgrades. 1) Grade J: Mostly independent and able to go outside. J1: can use public transport; J2: can visit neighbors without public transport. 2) Grade A: Mostly independent indoors. A1: goes out with assistance; A2: rarely goes out and alternates between bed and other areas. 3) Grade B: Requires assistance indoors, usually bedridden but can maintain a seated position. B1: able to eat and use the toilet independently with a wheelchair; B2: requires help to use a wheelchair. 4) Grade C: Completely bedridden and requires full assistance. C1: can roll over; C2: cannot roll over.

Dementia severity is classified into five grades: I, II (IIa, IIb), III (IIIa, IIIb), IV, and M. Grade I: Mild dementia, independent in daily life. Grade II: Symptoms affect daily life; IIa: observed outside the home; IIb: observed at home. Grade III: More severe symptoms; IIIa: daytime, IIIb: nighttime. Grade IV: Frequent symptoms requiring constant care. Grade M: Severe psychiatric symptoms or comorbidities requiring specialized care.

All bedridden and dementia classifications were determined by the attending physician.

Statistical analysis

Based on changes before and after home-visit rehabilitation in BI, FAI, care-need levels, and ratings for bedridden status and dementia, participants were categorized into three subgroups:

Improvement-sustenance covers those who showed meaningful improvement, defined for the purposes of this study as a BI increase of ≥5 points or a reduced care-need level, and maintained it.

Improvement-aggravation covers those who initially improved but later declined below baseline levels.

Aggravation covers those who showed continuous decline without initial improvement.

Categorical variables (e.g., sex, diseases, cohabitants, frequency/time of rehab) were analyzed using the chi-square test. The distribution of continuous variables (e.g., age, BI, FAI, care level) was assessed using the Shapiro-Wilk test, which indicated non-normality. Therefore, these variables were analyzed using the Mann-Whitney U test. Ordinal outcomes (e.g., care level, bedridden, and dementia ratings) were evaluated using Fisher’s exact test, with multiple comparisons adjusted using Holm’s step-down procedure. This method was consistently applied to all subgroup comparisons. Statistical analysis was performed using IBM Corp. Released 2020. IBM SPSS Statistics for Windows, Version 26. Armonk, NY: IBM Corp., and statistical significance was set at p < 0.05.

## Results

Patient characteristics

Table 1 presents the characteristics of participants in Groups A and B. A total of 156 individuals (71 men, 85 women) were assigned to Group A, while 56 individuals (25 men, 31 women) were assigned to Group B. The mean age ± standard deviation was 81.2 ± 9.2 years in Group A and 79.0 ± 11.4 years in Group B. A significantly higher proportion of participants in Group B received home-visit rehabilitation two or more times per week (χ² = 49.297, p < 0.001). No significant differences were found between the two groups in terms of other baseline characteristics.

**Table 1:** Patient characteristics Number (%), chi-square test †：average ± standard deviation (minimum-maximum)，Mann–Whitney U test

		Group A （n=156）			Group B （n=56）			p-value	
Age^†^		81.2 ± 9.2 (41 - 99)			79.0 ± 11.4 (43 - 94)			0.433	
Sex（men/women）		71 (45)	/	85 (54)	25 (44)	/	31 (55)	0.911	
Underlying diseases（+/-）	Malignancy	145 (92)	/	11 (7)	51 (91)	/	5 (8)	0.658	
	Motor function disease	94 (60)	/	62 (39)	34 (60)	/	22 (39)	0.952	
	Respiratory disease	149 (95)	/	7 (4)	55 (98)	/	1 (1)	0.363	
	Cardiovascular disease	147 (94)	/	9 (5)	52 (92)	/	4 (7)	0.713	
	Cerebrovascular disease	128 (82)	/	28 (17)	41 (73)	/	15 (26)	0.158	
	Disuse syndrome	146 (93)	/	10 (6)	51 (91)	/	5 (8)	0.528	
	Neuromotor disease	141 (90)	/	15 (9)	55 (98)	/	1 (1)	0.057	
	Visceral disease	149 (95)	/	7 (4)	53(94)	/	3 (5)	0.792	
	Dementia	150 (96)	/	6 (3)	56 (100)	/	0 (0)	0.137	
	Mental illness	155 (99)	/	1 (0)	56 (100)	/	0 (0)	0.548	
Cohabitants（+/-）		112 (71)	/	44 (28)	44 (78)	/	12 (21)	0.324	
Spouse （+/-）		79 (50)	/	77 (49)	29 (51)	/	27 (48)	0.883	
Relatives （+/-）		52 (33)	/	104 (66)	22 (39)	/	34 (60)	0.423	
Home-visit rehabilitation-	Frequency（once a week / two or more）	127 (81)	/	29 (18)	17 (30)	/	39 (69)	< 0.001	
	Rehabilitation time （40 minutes / 60 minutes）	154 (98)	/	2 (1)	56 (100)	/	0 (0)	0.395	

Changes in the Barthel index and the Frenchay activities index scores

Table 2 summarizes the BI and FAI scores before and after home-visit rehabilitation. Compared to Group A, Group B showed significantly lower initial FAI scores (U = 3579, p = 0.038), as well as significantly greater improvements in BI scores (U = 3375.5, p = 0.001) and FAI scores (U = 3618.5, p = 0.028). Table 3 shows the distribution of clinical outcomes in BI and FAI. The proportion of participants classified in the BI improvement-sustenance subgroup was significantly higher in Group B (Fisher’s exact test, p < 0.001). No significant differences were observed in other outcome variables related to BI and FAI.

**Table 2:** Comparison of Barthel index and Frenchay activities index scores between group A and group B before and after home-visit rehabilitation Mean ± standard deviation, Range（minimum-maximum)，Mann-Whitney’s U-test

		Group A (n=156)		Group B (n=56)		
Measure		Mean ± SD	Range	Mean ± SD	Range	p-value
Barthel Index	Initial	65.87 ± 30.90	0–100	58.57 ± 31.79	0–100	0.149
	Six months / termination	65.58 ± 31.89	0–100	61.52 ± 32.32	0–100	0.529
	Change	-0.29 ± 8.02	-65–20	2.95 ± 10.86	-50–35	0.001
Frenchay Activities Index	Initial	6.71 ± 8.04	0–31	4.25 ± 6.50	0–29	0.038
	Six months / termination	7.62 ± 8.40	0–31	6.48 ± 7.49	0–29	0.419
	Change	0.91 ± 2.67	-6–15	2.23 ± 4.54	-9–19	0.028

**Table 3:** Comparison of clinical outcome distributions (improvement, sustenance, aggravation) in the Barthel index and Frenchay activities index between group A and group B Number (%), Fisher’s exact test (multiple comparison procedure: Holm’s step-down procedure), α: improvement/sustenance × group A/group B (p < 0.05)

		Group A (n=156)	Group B (n=56)		
Clinical outcome		n (%)	n (%)	p-value	Multiple comparison
Barthel Index	Improvement	19 (12%)	20 (35%)	<0.001	α
	Sustenance	125 (80%)	32 (57%)		
	Aggravation	12 (7%)	4 (7%)		
Frenchay Activities Index	Improvement	42 (26%)	22 (39%)	0.085	-
	Sustenance	101 (64%)	31 (55%)		
	Aggravation	13 (8%)	3 (5%)		

Changes in care-need level, bedridden status, and dementia rating

Table 4 displays the results of changes in care-need level, bedridden status, and dementia rating between the two groups. Group B had significantly higher initial care-need levels (U = 3230.5, p = 0.003) and bedridden status (U = 3520.5, p = 0.028) than Group A. Table 5 shows the distribution of clinical outcomes in these measures. The proportion of participants in the improvement-sustenance subgroup for bedridden status was significantly higher in Group B (Fisher’s exact test, p = 0.005). No statistically significant differences were found between the two groups regarding changes in dementia rating or other related variables.

**Table 4:** Comparison of care level, bedridden degree, and dementia rating scores between group A and group B before and after home-visit rehabilitation Mean ± standard deviation, Range（minimum-maximum)，Mann-Whitney’s U-test, levels of support or long-term care needed (1: level of support needed 1, 2: level of support needed 2, 3: level of long-term care needed 1, 4: level of long-term care needed 2, 5: level of long-term care needed 3, 6: level of long-term care needed 4, and 7: level of long-term care needed 5), degree of being bedridden (0: independent, 1: J1, 2: J2, 3: A1, 4: A2, 5:B1, 6:B2, 7: C1, and 8: C2), dementia rating (0: independent, 1: Ⅰ, 2: Ⅱa, 3: Ⅱb, 4: Ⅲa, 5: Ⅲb, 6: Ⅳ, and 7: M)

		Group A (n=156)		Group B (n=56)		
Measure		Mean ± SD	Range	Mean ± SD	Range	p-value
Levels of support or long-term care needed	Initial	4.00 ± 1.79	1–7	4.80 ± 1.83	1–7	0.003
	Six months / termination	4.31 ± 1.66	1–7	4.75 ± 1.85	1–7	0.07
Degree of being bedridden	Initial	4.07 ± 1.82	0–8	4.71 ± 1.81	1–8	0.028
	Six months / termination	4.13 ± 1.89	0–8	4.46 ± 2.18	1–8	0.335
Dementia rating	Initial	1.69 ± 1.80	0–7	1.32 ± 1.49	0–6	0.228
	Six months / termination	1.79 ± 1.85	0–7	1.32 ± 1.39	0–6	0.179

**Table 5:** Comparison of clinical outcome distributions (improvement, sustenance, aggravation) of care level, bedridden degree, and dementia rating between group A and group B Number (%), Fisher’s exact test (multiple comparison procedure: Holm’s step-down procedure), levels of support or long-term care needed (1: level of support needed 1, 2: level of support needed 2, 3: level of long-term care needed 1, 4: level of long-term care needed 2, 5: level of long-term care needed 3, 6: level of long-term care needed 4 and 7: level of long-term care needed 5), degree of being bedridden (0: independent, 1: J1, 2: J2, 3: A1, 4: A2, 5:B1, 6:B2, 7: C1, and 8: C2), dementia rating (0: independent, 1: Ⅰ, 2: Ⅱa, 3: Ⅱb, 4: Ⅲa, 5: Ⅲb, 6: Ⅳ, and 7: M), α: improvement/sustenance × group A/group B (p < 0.05), n.s.: not significant

		Group A (n=156)	Group B (n=56)		
Clinical outcome		n (%)	n (%)	p-value	Multiple comparison
Levels of support or long-term care needed	Improvement	3 (1%)	3 (5%)	0.054	-
	Sustenance	123 (78%)	49 (87%)		
	Aggravation	30 (19%)	4 (7%)		
Degree of being bedridden	Improvement	16 (10%)	16 (28%)	0.005	α
	Sustenance	122 (78%)	33 (58%)		
	Aggravation	18 (11%)	7 (12%)		
Dementia rating	Improvement	11 (7%)	10 (17%)	0.042	n.s.
	Sustenance	128 (82%)	38 (67%)		
	Aggravation	17 (10%)	8 (14%)		

## Discussion

This study investigated how the timing of initiating home-visit rehabilitation influences improvements in ADLs and IADLs among older adults. The results showed that older adults in Group B, who began rehabilitation soon after discharge, had significantly higher initial care-need levels and bedridden status and lower initial FAI scores compared to those in Group A. However, this finding must be interpreted with extreme caution, as Group B also received home-visit rehabilitation with significantly higher frequency. This difference in rehabilitation intensity represents a major confounding factor, making it impossible to disentangle the effect of initiation timing from that of the intervention dosage. Therefore, our findings do not establish a causal relationship but rather present an exploratory observation from a real-world clinical setting, highlighting a complex interplay between patient characteristics, timing, and intensity of care.

In our study, the number of older adults in Group B who received home-visit rehabilitation two or more times per week was significantly higher than in Group A (Table 1). Kamioka et al. examined the total monthly duration of home-visit rehabilitation and found it to be 315.5 minutes for individuals within one year of onset, 252.0 minutes for those between one and three years, and 237.9 minutes for those over three years since onset [4]. In our study, participants in Group B typically received 40-minute sessions twice or more per week, approximately 320 minutes monthly, consistent with the findings of Kamioka et al. Although we did not investigate the exact clinical onset for each participant, individuals discharged from hospitals or nursing facilities typically began rehabilitation after an acute event or exacerbation of a chronic condition. As in the previous study, those in the earlier stages of illness or recovery were more likely to receive frequent home-visit rehabilitation. The increased frequency in Group B may also reflect concerns about functional decline [9] and the need to reduce caregiver burden [10].

Regarding changes in BI and FAI scores, Group B showed significantly greater improvements in both indices and a significantly higher proportion of individuals in the improvement-sustenance subgroup for BI (Table 2). Previous studies have reported that early initiation of home-visit rehabilitation can improve ADLs [4,11]. The average age of Group B participants in our study was slightly higher than those in previous studies [4,11], and unlike earlier research, we categorized underlying conditions into 10 distinct groups. Despite this heterogeneity, improvements consistent with previous findings were observed.

Initial FAI scores in Group B were significantly lower than those in Group A. Nomura et al. reported a reduction in IADL training frequency after discharge from a rehabilitation ward compared to during hospitalization [12]. Although the participants in our study were not recruited from rehabilitation wards, they also showed reduced IADL levels after discharge. This suggests that IADLs may decline soon after discharge, possibly due to decreased ADLs. Ishida et al. found IADL improvements among older adults with acute conditions such as stroke or fractures [5]. While our study included a broader range of diagnoses, Group B still exhibited significantly greater FAI score improvements, reinforcing the potential for IADL gains even among more diverse populations.

Clinically, the 2.95-point improvement in BI observed in Group B exceeds the established minimal clinically important difference (MCID) of 1.8 points, indicating a meaningful enhancement in ADLs [13]. Although no definitive MCID exists for the FAI, the 2.23-point improvement observed suggests a potentially meaningful change in IADLs that warrants further study [7].

Group B also exhibited significantly higher initial care-need levels and bedridden status (Table 4). In Japanese clinical practice, care certification is often renewed during hospital stays due to acute functional decline. As such, the elevated care levels and bedridden status in Group B likely reflect recent hospitalizations and underlying disease progression [14,15]. Notably, the proportion of participants in the improvement-sustenance subgroup for bedridden status was significantly higher in Group B. To our knowledge, few prior studies have explored this outcome. Previous reports suggest that early initiation of home-visit rehabilitation can improve ADLs, IADLs, and bedridden status [4,5,11,16]. The observed improvements in Group B may reflect gains in both physical and mental function through timely intervention.

In summary, this study observed that initiating rehabilitation soon after discharge was associated with greater functional improvement; however, this association was heavily confounded by a higher frequency of intervention in the post-discharge group. It remains unclear whether the observed benefits are attributable to the timing of initiation, the greater intensity of the rehabilitation, a combination of both, or other unmeasured patient factors such as motivation or family support. This ambiguity underscores the need for more rigorous research. Rather than emphasizing the clinical importance of early initiation, our findings highlight that the post-discharge period is often a time of more intensive intervention, which is associated with better outcomes. Facilitating a smooth and intensive transition to homecare requires strengthened collaboration between medical and long-term care professionals.

Limitations

This study has several significant limitations that must be acknowledged when interpreting the findings. First and foremost, the primary limitation is the significant confounding by indication and intervention intensity. Our results showed that Group B, who began rehabilitation after discharge, received substantially more frequent therapy than Group A. This clinical reality, where patients with more acute needs receive more intensive care, makes it impossible to disentangle the effects of initiation timing from the effects of rehabilitation dosage. Furthermore, the non-randomized, retrospective design introduces a high risk of selection bias. A host of unmeasured confounders, including specific disease severity, cognitive function, patient motivation, caregiver availability, and socioeconomic status, likely influenced both group assignment and outcomes. Collectively, these factors make any causal inference between timing and improvement impossible. Second, the study's design limits the generalizability of its findings. As a retrospective analysis conducted at a single institution, the results may not be applicable to other clinical settings. Clinical outcomes and rehabilitation protocols can differ significantly between institutions and even across different regions within Japan, as highlighted by Futohashi et al. [17]. Furthermore, the services were provided under the specific framework of Japan's Long-Term Care Insurance system, and the findings may not translate to countries with different models of post-acute care and discharge planning. Third, several potential sources of information bias and measurement limitations exist. We did not analyze the characteristics of patients who discontinued rehabilitation or the reasons for their discontinuation, which introduces a risk of attrition bias. The outcomes may systematically differ between those who completed the observation period and those who did not. Additionally, the metrics used for improvement have limitations. The use of an MCID for the Barthel Index derived from stroke patients may not be entirely appropriate for our diagnostically heterogeneous population. Similarly, the threshold for meaningful improvement (a 5-point change in the BI) was established for practical purposes and lacks a specific evidence base for this population. Given these substantial limitations, our findings should be considered strictly exploratory and hypothesis-generating, rather than conclusive evidence supporting a specific intervention strategy.

## Conclusions

In this exploratory retrospective study, we observed an association between the timing of home-visit rehabilitation initiation and functional outcomes. Older adults who began rehabilitation soon after discharge tended to receive more frequent therapy and, in turn, showed greater improvements in ADLs and IADLs compared to those who started rehabilitation while already living at home. However, due to significant confounding by rehabilitation intensity and baseline patient characteristics, these findings do not establish a causal link between earlier initiation timing and better outcomes. The observed improvements in the post-discharge group could be largely attributable to the higher dosage of therapy they received. Therefore, we conclude not that early timing itself is superior, but that the post-discharge period may represent a critical window where patients are receptive to, and are provided with, more intensive rehabilitation that is associated with functional gains. Our findings should be considered hypothesis-generating. Future prospective studies, ideally with designs that can control for intervention dose and patient selection, are required to isolate the independent effects of timing and intensity on rehabilitation outcomes.
